# Development and antimicrobial susceptibility studies of in vitro monomicrobial and polymicrobial biofilm models with *Aspergillus fumigatus* and *Pseudomonas aeruginosa*

**DOI:** 10.1186/1471-2180-14-53

**Published:** 2014-03-03

**Authors:** Elias K Manavathu, Dora L Vager, Jose A Vazquez

**Affiliations:** 1 Henry Ford Hospital, 2799 West Grand Boulevard, Detroit, Michigan 48202, USA; 2 Current address: Division of Infectious Diseases, Department of Medicine, Georgia Regents University, 1120 15th Street, AE 3029, Augusta, GA 30912, USA

## Abstract

**Background:**

Mixed microbial infections of the respiratory tracts with *P. aeruginosa* and *A. fumigatus* capable of producing biofilms are commonly found in cystic fibrosis patients. The primary objective of this study was to develop an *in vitro* model for *P. aeruginosa* and *A. fumigatus* polymicrobial biofilm to study the efficacy of various antimicrobial drugs alone and in combinations against biofilm-embedded cells. Simultaneous static cocultures of *P. aeruginosa* and sporelings were used for the development of in vitro *P. aeruginosa*-*A. fumigatus* polymicrobial biofilm in SD broth in 24-well cell culture plates at 35°C, and the biofilm formation was monitored microscopically and spectrophotometrically. Using *P. aeruginosa*-*A. fumigatus* sporelings cocultures we examined the effects of various antimicrobial drugs alone and in combination against polymicrobial biofilm by CFU and tetrazolium reduction assays.

**Results:**

In simultaneous static cocultures *P. aeruginosa* cells killed *A. fumigatus* conidia, whereas the bacterial cells showed no substantial fungicidal effect on sporelings grown for 12 h or longer at 35°C. Monospecies cultures of *P. aeruginosa* produced loosely adhered monomicrobial biofilm and addition of 10% bovine serum to the growth medium inhibited the formation of monomicrobial biofilm by *P. aeruginosa* whereas it produced tightly adhered polymicrobial biofilm in the presence of *A. fumigatus* mycelial growth. *A. fumigatus* produced firmly adherent monomicrobial and polymicrobial biofilms. A comparison of CFU and MTT assays showed that the latter is unsuitable for studying the effectiveness of antimicrobial treatment against polymicrobial biofilm. Tobramycin alone and in combination with posaconazole was highly effective against monomicrobial and polymicrobial biofilms of *P. aeruginosa* whereas cefepime alone and in combination with posaconazole showed excellent activity against monomicrobial biofilm of *P. aeruginosa* but was less effective against polymicrobial biofilm. Monomicrobial and polymicrobial biofilms of *A. fumigatus* showed similar susceptibility to posaconazole with and without the antibacterial drug.

**Conclusions:**

Simultaneous static coculture of *A. fumigatus* sporelings grown for 12 h or longer was superior to ungerminated conidia with *P. aeruginosa* for the development of *A. fumigatus*-*P. aeruginosa* biofilm. *P. aeruginosa*-*A. fumigatus* polymicrobial biofilm shows differential susceptibility to antimicrobial drugs whereas the susceptibility of *A. fumigatus* to antimicrobial drugs was unchanged.

## Background

Polymicrobial infection caused by multiple species of microorganisms belonging to markedly different taxonomic groups is a common occurrence in severely immunocompromised patients [1-5] as well as in individuals suffering from persistent diabetic wounds [6-9], chronic pulmonary obstructive disease [10-13], cystic fibrosis patients suffering from chronic infections [14-20] and lung transplant recipients [21-23]. The microorganisms more commonly isolated from mixed microbial infections are pathogenic bacteria and fungi. A recent retrospective study of the respiratory tract microbiology of cystic fibrosis patients revealed that their airways were colonized by multiple microorganisms, in particular *Pseudomonas aeruginosa* (62% prevalence) in association with *Aspergillus* species [24]. The epidemiology and clinical significance of *Aspergillus* infection in cystic fibrosis patients have been recently reviewed [25-27]. Among the numerous *Aspergillus* isolates recovered from the respiratory tracts of cystic fibrosis patients, *A. fumigatus* is the most predominant species with a prevalence ranging from 11% to 14% in the United States [28] and as high as 60% to 78% in Europe [29,30], followed by *A. terreus*. Although invasive aspergillosis can occur in persons with cystic fibrosis, particularly after lung transplantation, the most common complication of *Aspergillus* infection is allergic bronchopulmonary aspergillosis [31-34], a condition that causes the deterioration of lung function associated with wheezing, shortness of breath, cough and chest pain.

Given the high prevalence of *P. aeruginosa* and *A. fumigatus* colonization of the airways of cystic fibrosis patients, mixed microbial infection involving these microorganisms commonly occurs in the lungs [30,35,36] producing monomicrobial and polymicrobial biofilms. The biofilm-embedded cells are highly resistant to antimicrobial drug therapy [37-40], difficult to eradicate and often develop chronic infection that acts as a reservoir causing serious life-threatening infection in individuals with debilitated immune function. Several investigators have recently studied *A. fumigatus* monomicrobial biofilm using in vitro [40] and human bronchial epithelial cell culture [38] models. The aerial or surface biofilm is similar to the fungal ball often associated with aspergilloma in patients with lung cavitary lesions. The aerial biofilm made up of fungal mycelia bound together by an extracellular matrix composed of a variety of macromolecules, including galactomannan, α1,3-glucan, monosaccharides and polyols, melanin, proteins including major antigens and hydrophobin molecules [41]. On the other hand, Loussert et al. have recently [42] studied the composition of the mycelial extracellular matrix in vivo and found to have less complex but similar composition. The monomicrobial biofilm of *A. fumigatus* developed in 96-well cell culture plates and in human bronchial epithelial cell culture were resistant to antimicrobial drugs [38,40]. Gene expression and proteomic studies by Bruns et al. [43] showed that the 24-h biofilm expressed a greater variety of genes whereas more mature older biofilm expressed mainly specialized genes for the synthesis of extracellular matrix and secondary metabolites such as gliotoxin. Mowat *et al*[44] and Moree *et al*[45] have recently investigated the in vitro interaction of *A. fumigatus* with *P. aeruginosa* and demonstrated that *A. fumigatus* biofilm formation is inhibited by small diffusible molecules produced by *P. aeruginosa* whereas preformed biofilm was only mildly affected. To date, very little is known about the characteristics and antimicrobial drug susceptibility of mixed microbial biofilm produced by *A. fumigatus* and *P. aeruginosa*. In this paper we describe the development and antimicrobial drug susceptibility of a simple highly reliable in vitro polymicrobial biofilm model for *A. fumigatus* and *P. aeruginosa* in 24-well cell culture plates using cocultures.

## Methods

### Microorganisms and culture conditions

*A. fumigatus* 53470 (AF53470), *A. fumigatus* ATCC36607 (AF36607), *P. aeruginosa* 56402 (PA56402) and *P. aeruginosa* ATCC27853 (PA27853) were used in this study. AF53470 and PA56402 were clinical isolates obtained from the Microbiology Laboratory of Henry Ford Hospital in Detroit, Michigan, USA whereas AF36607 and PA27853 were commercially obtained from the American Type Culture Collection, Manassas, VA 20110**,** USA.

The initial AF53470 and AF36607 cultures obtained from the Microbiology Laboratory and American Type Culture Collection were subcultured on SD agar (Difco brand, Becton Dickenson Diagnostics, Sparks, MD 21152, USA) for checking the viability and purity, and subsequently stored as conidial suspension in 25% glycerol at -80°C. Working cultures were routinely maintained on SD agar plates at 4°C. AF53470 and AF36607 were highly susceptible to polyenes, triazoles and echinocandins, including amphotericin B, voriconazole, posaconazole (MICs 1 μg/ml, 0.25 μg/ml, 0.062 μg/ml, respectively) and anidulafungin (MEC 0.031 μg/ml). For preparation of conidia, cultures were grown on SD agar plates for 4 days at 35°C to produce large amount of conidia. The SD agar containing the mycelial growth was cut into small (5 mm^2^) pieces using a sterile spatula, transferred to a 50-ml screw-capped conical culture tube containing 25 ml sterile distilled water and vortexed vigorously for 2 min to disperse the conidia from the conidiophores. The resulting fungal suspension was filtered through 8 layers of sterile cheese cloth to remove mycelial and agar debris. The clarified conidial suspension thus obtained was standardized by hemocytometer count and stored at 4°C in the refrigerator. *A. fumigatus* conidia do not germinate in sterile distilled water at 4°C in the refrigerator and remain viable for several months, thus if required the same batch of conidial suspension can be used for several experiments.

The initial PA56402 and PA27853 cultures obtained from the Microbiology Laboratory and American Type Culture Collection were subsequently subcultured on BHI agar (Difco brand, Becton Dickenson Diagnostics, Sparks, MD 21152, USA) for the evaluation of purity and viability. The colony purified isolates were stored in 25% glycerol at -80°C. Working cultures were routinely grown on BHI agar, stored at 4°C and subcultured at 37°C once a week to maintain viable stock cultures. PA56402 and PA27853 were highly susceptible to a variety of antibacterial drugs such as aminoglycosides, β-lactams and fluoroquinolones, including tobramycin (MIC 0.125 μg/ml), cefepime (MIC ≤1 μg/ml) and ciprofloxacin (MIC ≤ 0.25 μg/ml). Since PA56402 and PA27853 grew well in SD broth we used this medium for growing polymicrobial biofilms of *A. fumigatus* and *P. aeruginosa* in mixed cultures. One ml aliquots of the overnight cultures were centrifuged in a microcentrifuge at top speed for 2 min and the pellets were washed 3 times (1 ml each) with sterile distilled water, resuspended in 1 ml fresh SD broth, standardized spectrophotometrically using a standard curve and subsequently used for various experiments. The use of SD broth was particularly convenient for biofilm development since it was commonly used to grow *A. fumigatus* cultures.

### Biofilm development

For the development of *A. fumigatus* and *P. aeruginosa* monomicrobial and polymicrobial biofilm models, we used Costar 24-well flat bottom cell culture plates [Cat. no. 3526, Corning Incorporated, Corning, NY 14831, USA]. Briefly, 1 × 10^6^*A. fumigatus* conidia prepared as described above were incubated in 1 ml SD broth at 35°C in 24-well cell culture plates for 18 h, and allowed them to germinate and grow producing a tightly adherent monolayer of mycelial growth at the bottom of the well. The surface mycelial growth was removed using a sterile spatula and the spent growth medium was removed by aspiration with a 1-ml micropipet. The adherent mycelial layer was washed (3 times with sterile distilled water, 1 ml each) using a 1-ml micropipet and the wash fluid was completely removed by aspiration. One ml SD broth was added to the mycelial growth (18 h) and then inoculated with 1 × 10^6^*P. aeruginosa* cells. The mixed culture was incubated at 35°C for either 24 h or 48 h for the development of a mixed microbial culture producing polymicrobial biofilm. At the end of the coculturing period, any remaining surface mycelial growth was removed as previously described and the mixed fungal-bacterial culture adhered to the bottom of the 24-well tissue culture plate was washed three times with sterile distilled water (1 ml each). The adherent layer of fungal and bacterial cells was scraped with a wet sterile swab, resuspended in 1 ml of sterile distilled water, vortexed vigorously for 30 seconds with 0.1 g sterile glass beads to resuspend the cells and the biofilm growth was determined by CFU and tetrazolium reduction assays. For CFU assay, the cell suspensions were serially diluted 10 to 10^8^ fold and 0.01 ml aliquots were spotted on SD agar plates containing either ciprofloxacin (50 μg/ml) or voriconazole (16 μg/ml) for selective fungal and bacterial growth. The numbers of CFUs of *A. fumigatus* and *P. aeruginosa* were determined after 24 h growth at 35°C. For the development of monomicrobial biofilms, *A. fumigatus* conidia and *P. aeruginosa* cells were grown as monomicrobial cultures under identical conditions and assayed for fungal and bacterial CFUs.

### Photomicrography

**Figure 1:**
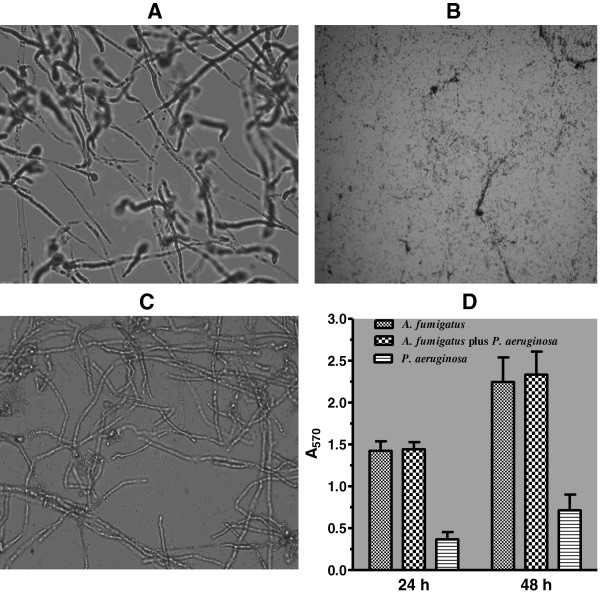
**Photomicrographic images and quantification of *A. fumigatus *and *P. aeruginosa *biofilms.A**. Monomicrobial biofilm of AF53470 grown on plastic cover slips for 48 h at 35°C. **B**. Monomicrobial biofilm of PA56402 grown on plastic cover slips for 48 h at 35°C. **C**. Polymicrobial biofilm formed in coculture by AF53470 sporelings and PA56402 grown on plastic cover slips for 48 h at 35°C. The biofilms were photographed using a Nikon Microscope Camera System equipped with SPOT image processing computer software [46]. With the SPOT program, each Objective (10× to 100×) of the microscope was calibrated using a stage micrometer as previously described in the SPOT Software User Guide (Chapter 4, pages 76 and 77). The photomicrographs shown in Figure 1 were captured using the 60× Objective providing a total magnification of 600×. **D**. Quantification of 24-h and 48-h monomicrobial and polymicrobial biofilms of AF53470 and PA56402. The biofilm quantification experiment by crystal violet binding assay was performed two times with eight replications for each group. The data were analyzed by two-way ANOVA and paired Student’s *t*-test using GraphPad Prism 5.0. The vertical bar on each histogram represents the standard error of the mean for two independent experiments. The laboratory isolates AF36607 and PA27853 also produced similar monomicrobial and polymicrobial biofilms on plastic cover slips and Costar 6-well cell culture plates.

For photomicrography the monomicrobial and polymicrobial biofilms of *A. fumigatus* and *P. aeruginosa* were grown either on 22 mm sterile plastic microscopic cover slips (Cat. no. 12547, Fisher Scientific Company, Pittsburgh, PA) or in Costar 6-well flat bottom cell culture plates [Cat. no. 3736, Corning Incorporated, Corning, NY 14831, USA] in SD broth at 35°C. Briefly, the sterile plastic cover slips were placed in a Costar 6-well cell culture plate. Three ml aliquots of the *A. fumigatus* conidial suspension containing 1 × 10^6^ conidia/ml were placed in each well completely covering the plastic cover slip and the cell culture plate was incubated statically at 35°C for 18 h for *A. fumigatus* conidia to germinate and form a monolayer of mycelial growth on the plastic cover slips. The spent growth medium from each well was removed and the cover slips containing the mycelial growth were washed (3 times with sterile distilled water, 2 ml each) and inoculated with 3 ml of SD broth containing 1 × 10^6^*P. aeruginosa* cells/ml. The mixed microbial culture was incubated for 24 h at 35°C for the development of *A. fumigatus-P. aeruginosa* polymicrobial biofilm. The plastic cover slips containing the mixed microbial growth were washed (3 times with sterile distilled water, 2 ml each) and transferred to a clean Costar 6-well cell culture plate and stained with crystal violet (0.04%) for 30 min at 35°C. The stained cover slips were washed (4 times with sterile distilled water, 2 ml each) and the excess water was drained. The cover slips were briefly air-dried, mounted on a standard microscopic slide using nail polish and the biofilms were photographed using a Nikon Microscope Camera System equipped with SPOT image processing computer software [46]. With the SPOT program, each Objective (10× to 100×) of the microscope was previously calibrated using a stage micrometer as described in the SPOT Software User Guide (Chapter 4, pages 76 and 77). The photomicrographs shown in Figure 1 were captured using the 60X Objective providing a total magnification of 600X. To develop monomicrobial biofilms of *A. fumigatus* and *P. aeruginosa*, monomicrobial cultures of these organisms were grown on plastic cover slips and processed identically. To study the kinetics of *A. fumigatus* monomicrobial biofilm development from conidia, monomicrobial cultures of *A. fumigatus* were grown in SD broth from a conidial suspension for 0 h to 24 h in Costar 6-well cell culture plates, washed, stained and photographed as described above.

### Determination of the effects of antibiotics on biofilms

Monomicrobial and polymicrobial biofilms of *A. fumigatus* and *P. aeruginosa* were developed in Costar 24-well cell culture plates as previously described. The biofilms were washed with distilled water (3 times, 1 ml each) and incubated with the appropriate concentrations of antimicrobial drug(s) for 24 h at 35°C. The drug-treated biofilms were washed and the adherent cultures containing either fungal or bacterial or a mixed population of fungal and bacterial cells were harvested by scraping the bottom of the wells of the cell culture plates using sterile wet swabs into 1 ml aliquots of sterile distilled water. The cell suspension was vortexed vigorously with sterile glass beads to disperse the cells, serially diluted 10 to 10^8^ fold and 0.01 ml aliquots of the cell suspensions were plated on ciprofloxacin (50 μg/ml) or voriconazole (16 μg/ml) containing SD agar plates and incubated for 24 h at 35°C for selective growth. The number of CFUs for each group was determined and plotted against the drug concentration to assess the effectiveness of antibiotic treatment against biofilm bound cells.

One of the disadvantages of using CFU assay to determine the growth of filamentous fungi is the poor correlation between biomass and CFU values. We therefore performed a pilot experiment where 1 × 10^6^ conidia were germinated in 24-well cell culture plates in 1 ml SD broth at 35°C form 0 h to 24 and the fungal growth was determined by CFU assay. The number of CFUs obtained was more or less correlated with the number of conidia, germinated conidia and sporelings grown for up to 12 h. But once the hyphae grew extensively producing a mycelial biomass the correlation is usually reached a plateau and remained unchanged because of the geometry of the fully grown mycelial biomass and the pluripotent nature of the vegetative hyphae. Thus, the CFU assay for mature hyphae is at best an under estimation of the total fungal biomass. Since our experiments were designed to compare untreated drug-free controls to drug-treated experimental groups, determination of the absolute fungal biomass was not essential for demonstrating comparative effect of the drug treatment.

### Tetrazolium reduction assay

In addition to CFU assay, we evaluated the effects of antimicrobial drugs on monomicrobial and polymicrobial biofilms of *A. fumigatus* and *P. aeruginosa* by the tetrazolium reduction assay [47,48]. Briefly, monomicrobial and polymicrobial biofilms of *A. fumigatus* and *P. aeruginosa* were washed three times with sterile distilled water (1 ml each) and the excess water was removed by aspiration with a 1 ml micropipet. The washed adherent biofilm was overlaid with 1 ml fresh SD broth containing 100 mM 3-(4,5-dimethyl-2-thiazolyl)-2,5-diphenyl-2H-tetrazolium bromide [MTT] and 0.2 mM menadione and incubated at 35°C for 3 h for the reduction of the tetrazolium compound. Under these conditions, the lightly yellowish MTT will be reduced to an insoluble blue tetrazolium salt accumulated within the mycelia. At the end of the incubation period, the growth medium containing MTT was removed and the biofilm was washed three times (1 ml each) with sterile distilled water, and intracellular insoluble tetrazolium salt was dissolved in 1 ml 70% ethanol containing 0.1 N HCl for 30 min at 35°C. The amount of intracellular tetrazolium salts was quantified spectrophotometrically by measuring the absorbance of the solution at 570 nm. The accumulation of tetrazolium salt by the reduction of MTT by cellular dehydrogenases is proportional to the number of viable cells present in the biofilm. The effectiveness of the antimicrobial drug treatment was assessed on the basis of diminished tetrazolium reduction.

### Antimicrobial drugs

Pharmaceutical grade cefepime (Sagent Pharmaceuticals, Schaumberg, IL, USA) and tobramycin pure powder were obtained from the Henry Ford Hospital Pharmacy and Sigma Chemical Company, St. Louis, USA, respectively. Stock solutions (1 mg/ml) of the antibiotics were prepared in sterile distilled water and stored as 0.25 ml aliquots at -20°C. Voriconazole and posaconazole were obtained from Pfizer Pharmaceuticals (New York, NY, USA) and Schering-Plough Research Institute, Kenilsworth, NJ, USA (now part of Merck), respectively. The triazoles were dissolved in dimethylsulfoxide to obtain a stock solution of 10 mg/ml and stored as 0.25-ml aliquots at -20°C. The frozen stocks of the antimicrobial drugs were thawed at room temperature and used within 24 h. Where it is applicable, comparable concentrations of dimethylsulfoxide were used as control to examine its effect on the growth of the organism.

### Statistical analysis

The data were analyzed by Student’s *t* test, one-way and two-way analysis of variance with Bonferroni’s Multiple Comparison Test using Graphpad Prism Version 5.0 for Windows (GraphPad Software, Inc., La Jolla, CA, USA). A *p* value ≤0.05 was considered significant. Details of each statistical test used are given in the corresponding figure legend.

## Results

### Germinated conidia are more suitable for polymicrobial biofilm formation

**Figure 2:**
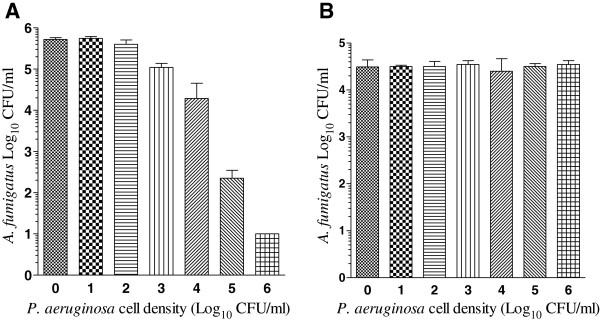
**Effects of *P. aeruginosa *on *A. fumigatus *conidia (A) and sporelings (B) in cocultures.***A. fumigatus* conidia **(A)** and sporelings **(B)** at a density of 1 × 10^6^ cells/ml were incubated with *P. aeruginosa* cells ranging from 1 x 10^1^-1 x 10^6^ cells/ml in 1 ml SD broth at 35°C for 24 h. At the end of the incubation the adherent microbial growth containing fungal and bacterial cells were washed 3 times with distilled water (1 ml each) and the viability of the cells was determined by CFU assay. In all mixed cultures the *P. aeruginosa* CFUs were similar (≈1 × 10^10^ CFU/ml). The experiment was performed at two different times with AF53470 and PA56402 using independently prepared conidial suspensions and bacterial cultures, and one time with AF36607 and PA27853. Similar results were obtained for the clinical and the laboratory isolates. The vertical bar on each data point represents the standard error of the mean for two independent experiments with AF53470 and PA56402. The data were analyzed by one way ANOVA with Dunnett multiple comparison test where the control was compared with each of the experimental group using GraphPad Prism 5.0.

The initial attempt for developing an in vitro *A. fumigatus*-*P. aeruginosa* polymicrobial biofilm model by simultaneous static coculturing of *A. fumigatus* conidia and *P. aeruginosa* cells at a cell ratio of 1:1 resulted in the complete killing of *A. fumigatus* cells. We therefore investigated the fungicidal effects of *P. aeruginosa* cell densities ranging from 1 × 10^1^ to 1 × 10^6^ cells/ml on the survival of 1 × 10^6^*A. fumigatus* conidia per ml after 24-h simultaneous static coculturing. As shown in Figure 2A, the fungicidal activity of *P. aeruginosa* against *A. fumigatus* conidia was directly proportional to *P. aeruginosa***:***A. fumigatus* cell ratio. Ten and hundred *P. aeruginosa* cells in 1 ml of SD broth containing 1 × 10^6^ conidia showed very little killing of *A. fumigatus* conidia (P = 0.5456 and 0.0871, respectively), 1 × 10^3^ and 1 × 10^4^*P. aeruginosa* cells showed moderate killing (P = 0.0002 and 0.0005, respectively) whereas 1 × 10^5^ and 1 × 10^6^*P. aeruginosa* cells killed *A. fumigatus* conidia 99.9% and 99.99% (P = 0.0003), respectively. In contrast, *P. aeruginosa* cell densities ranging from 1 × 10^1^-1 × 10^6^ cells/ml did not affect the viability of *A. fumigatus* sporelings grown from a conidial suspension for 12 h or longer and provided more or less the same number of CFU/ml [Figure 2B] after 24 h co-culturing. The lack of fungicidal activity was not because of *A. fumigatus* inhibition of *P. aeruginosa* growth since inoculation of sporelings with 1 × 10^1^ to 1 × 10^6^*P. aeruginosa* cells/ml provided approximately 1 × 10^10^*P. aeruginosa* CFU/ml indicating that growth of *P. aeruginosa* was not affected by the presence of 1 × 10^6^*A. fumigatus* sporelings/ml. The *P. aeruginosa* cells with faster growth rate reached stationary phase in 24 h in the presence of *A. fumigatus* sporelings and formed a polymicrobial biofilm suggesting that a range of *P. aeruginosa* cell densities could be used to develop a polymicrobial biofilm with *A. fumigatus* sporelings.

### Optimum conidial density for polymicrobial biofilm formation

**Figure 3:**
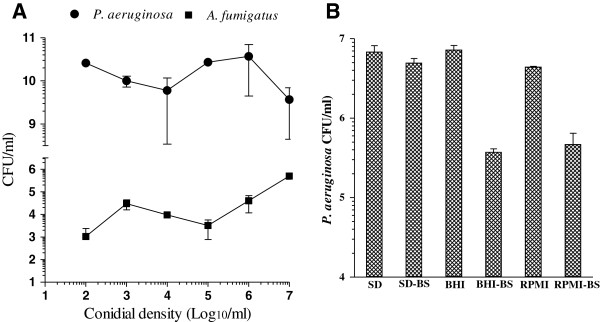
**Effects of cell density and growth medium on biofilm formation. A**. Effect of conidial density on *A. fumigatus*-*P. aeruginosa* polymicrobial biofilm formation. One ml aliquots of AF53470 conidial suspension containing 1 × 10^2^ - 1 × 10^7^ conidia/ml were incubated in 24-well cell culture plates in duplicates at 35°C in SD broth for 18 h, washed and then inoculated with 1 × 10^6^ PA56402 cells in 1 ml SD broth and further incubated for 24 h for the development of *A. fumigatus*-*P. aeruginosa* polymicrobial biofilm. The biofilm was washed and the embedded cells were resuspended in 1 ml sterile water and assayed for *A. fumigatus* and *P. aeruginosa* by CFU counts. The experiment was performed at two different times using independently prepared conidial suspensions and bacterial cultures and the vertical bar on each data point on the graph represents the standard error of the mean. **B**. *P. aeruginosa* monomicrobial biofilm formation in various growth media with and without bovine serum. One ml aliquots of growth media containing 1 × 10^6^*P. aeruginosa* cells were incubated in quadruplicates in 24-well cell culture plates with and without 10% bovine serum for 24 h at 35°C for biofilm formation. The adherent monomicrobial biofilm was washed (3 times), resuspended in 1 ml sterile distilled water and the biofilm growth was assessed by CFU assay. The experiment was performed two different times with PA56402 using independently prepared bacterial cultures, and one time with PA27853. Both sets of isolates provided similar results. The data were analyzed by paired Student’s *t* test using GraphPad prism 5.0. The vertical bar on each histogram denotes standard error of the mean for two independent experiments using PA56402. **Legends:** SD, Sabouraud’s dextrose broth; SD-BS, Sabouraud’s dextrose broth with 10% bovine serum; BHI, Brain Heart Infusion broth; BHI-BS Brain Heart Infusion broth with 10% bovine serum; RPMI, RPMI640; RPMI-BS, RPMI1640 with 10% bovine serum.

It was previously shown that *A. fumigatus* monomicrobial biofilm formation is a function of the conidial density and production of optimum amount of biofilm was dependent on the conidial density used [40]. We therefore examined the effect of conidial density on the development of *A. fumigatus*-*P. aeruginosa* polymicrobial biofilm. As shown in Figure 3A, a plot of *A. fumigatus* conidial density ranging from 1 × 10^2^ to 1 × 10^7^ conidia/ml used for the mycelial growth against the biofilm associated CFUs obtained for *A. fumigatus* and *P. aeruginosa* showed that a seeding density of 1 × 10^6^ conidia/ml provided the best yield of mixed microbial biofilm producing the most number of CFUs for both organisms. Although 1 × 10^7^conidia/ml produced the highest number of CFUs for *A. fumigatus*, the number of *P. aeruginosa* CFUs obtained was lower than that obtained when 1 × 10^6^conidia/ml was used. Among three different conidial densities (1 × 10^4^, 1 × 10^5^ and 1 × 10^6^ cells/ml) Mowat *et al.* used, 1 × 10^5^ conidia/ml produced the best *A. fumigatus* biofilm in a 96-well microtiter plate [36]. The difference may be due to the difference in the surface area of the wells of 96-well and 24-well cell culture plates, or the growth media (RPMI1640 vs. SD broth) used or the assays (tetrazolium reduction vs. CFU determination) used to measure the biofilm growth.

### Effects of various growth media with and without bovine serum on biofilm development

One of the primary objectives of this experiment was to identify a simple growth medium in which both *A. fumigatus* and *P. aeruginosa* would grow well and methodology for the formation of monomicrobial and polymicrobial biofilms will be simple for antimicrobial drug susceptibility testing of biofilms. The need to identify a suitable growth medium for *P. aeruginosa* biofilm formation was important because in general it produced poor monomicrobial biofilm on plastic surfaces such as polystyrene culture plates. Since pretreatment of certain plastics with bovine serum preconditions their surfaces for better cell attachment and biofilm production [49,50], we examined the effect of 10% bovine serum in the growth medium on the formation of *P. aeruginosa* biofilm. All three media we used were able to support the formation of *P. aeruginosa* biofilm to varying degree where BHI being the best medium followed by SD broth and RPMI1640 (Figure 3B). A comparison of the CFUs obtained for various media with and without bovine serum showed that the presence of 10% bovine serum inhibited *P. aeruginosa* monomicrobial biofilm formation by 27% in SD (P = 0.0509), 95% in BHI (P = 0.00016) and 89% in RPMI1640 (P = 0.00078) suggesting that bovine serum has a negative effect on *P. aeruginosa* biofilm formation in Costar cell culture plates. Thus, in our subsequent experiments, we used SD broth for the development of monomicrobial and polymicrobial biofilms of *A. fumigatus* and *P. aeruginosa*. The fact that *A. fumigatus* produces excellent monomicrobial biofilm in SD broth made it a highly suitable medium for the production of polymicrobial biofilms.

### Biofilm images and quantification

Figure 1 shows photomicrographic images of 24-h monomicrobial biofilms of *A. fumigatus* (A), *P. aeruginosa* (B) and *A. fumigatus-P. aeruginosa* polymicrobial biofilm (C) grown on plastic cover slips. *A. fumigatus* produced an extensive firmly adherent mycelial growth on the plastic cover slips and in any one microscopic field only a few hyphal filaments were in focus suggesting that as the hyphae grew they branched extensively forming a network of mycelial growth producing a three dimensional structure. The monomicrobial culture of *P. aeruginosa* growing on plastic cover slips formed a loosely adhered biofilm and gentle washing did not affect its stability on the plastic cover slips. On the other hand, washing of the biofilm with agitation randomly dislodged the cells from the plastic cover slips. The mixed microbial biofilm of *A. fumigatus* and *P. aeruginosa* showed a hazy background in which numerous *P. aeruginosa* cells were embedded in a mesh-like material. In the same planar field where the bacterial cells were in clear view the fungal hyphae were out of focus and numerous bacterial cells were seen adhered to the fungal hyphae using as scaffolding forming a mixed community of microbial growth.

Since the biofilm formation is known to increase with the duration of culturing, we investigated the effect of incubation time on the production of monomicrobial and polymicrobial biofilms of *A. fumigatus* and *P. aeruginosa.* A comparison of the amounts of crystal violet bound by 24-h and 48-h monomicrobial and polymicrobial biofilms of *A. fumigatus* and *P. aeruginosa* showed that the 48 h biofilm mass was increased by 57.7%, 61.7% and 94.5% (P ≤ 0.0044) for *A. fumigatus*, *A. fumigatus-P. aeruginosa* and *P. aeruginosa* biofilms, respectively (Figure 1D). However, no significant difference in CFUs was obtained for 24-h and 48-h biofilms (data not shown) suggesting that CFU determination is less than suitable for the determination fungal growth in more mature biofilms (e.g., 48 h biofilm). However, the 24 h and 48 h polymicrobial biofilms of *A. fumigatus-P. aeruginosa* were almost equally susceptible to antimicrobial drugs.

### Drug susceptibility studies

**Figure 4:**
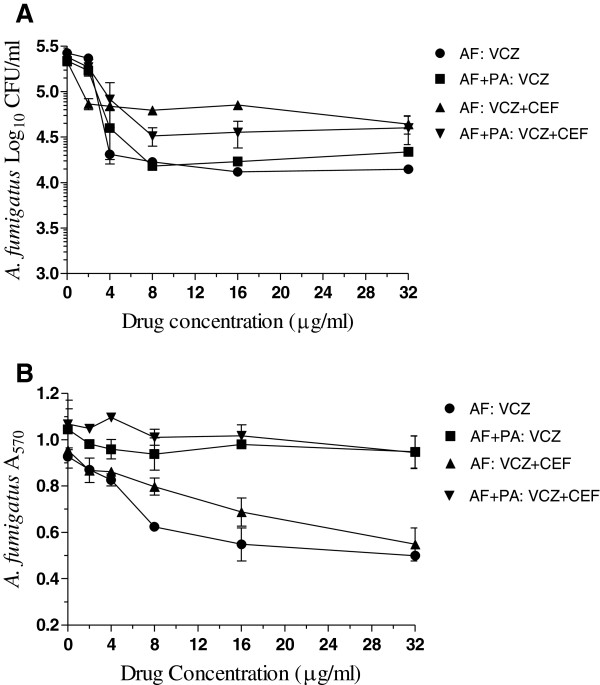
**Effects of voriconazole alone and in combination with cefepime against *A. fumigatus *monomicrobial and *A. fumigatus*-*P. aeruginosa *polymicrobial biofilms as determined by CFU (A) and MTT (B) assays.** The biofilms were developed in 24-well cell culture plates and the effectiveness of antimicrobial drug(s) treatment was assessed by the reduction of CFUs and A_570_ values. Each experiment was performed two different times with the clinical isolates AF53470 and PA56402 using independently prepared conidial suspensions and bacterial cultures, and one time with the laboratory isolates AF36607 and PA27853. Similar results were obtained for both set of isolates. The data were analyzed by two-way ANOVA with Bonferroni post test analysis by comparing each treatment group to the other for statistical significance using Graphpad Prism 5.0. The vertical bar on each data point denotes standard error of the mean for two experiments performed with AF53470 and PA56402. **Legends: AF**, *A. fumigatus* monomicrobial biofilm; **AF + PA**, *A. fumigatus-P. aeruginosa* polymicrobial biofilm; **VCZ**, voriconazole; **CEF**, cefepime.

To examine the suitability of our in vitro biofilm model for functional studies, we investigated the effectiveness of several antimicrobial drugs individually and in two-drug combinations against monomicrobial and polymicrobial biofilms of *P. aeruginosa* and *A. fumigatus* using CFU and tetrazolium reduction assays. Figure 4A shows representative results for voriconazole alone and in combination with cefepime on *A. fumigatus* monomicrobial and *A. fumigatus*-*P. aeruginosa* polymicrobial biofilms as determined by the CFU assay. Voriconazole at a concentration of 32 μg/ml reduced the CFU of monomicrobial and polymicrobial biofilms by approximately 1.5 logs suggesting that *A. fumigatus* cells embedded in monomicrobial and polymicrobial extracellular matrix were similarly susceptible (P = 0.3681) to the triazole voriconazole. On the other hand, voriconazole in combination with cefepime had slightly reduced antimicrobial activity against monomicrobial and polymicrobial biofilms (0.5 to 1 logs CFU reduction at 32 μg/ml) compared to voriconazole alone but showed no statistical significance (P = 0.5724).

Figure 4B shows the effectiveness of voriconazole alone and in combination with cefepime against *A. fumigatus* monomicrobial and *A. fumigatus*-*P. aeruginosa* polymicrobial biofilms as determined by MTT assay. A comparison of the A_570_ values obtained for monomicrobial and polymicrobial biofilms as a function of voriconazole concentration showed that the polymicrobial biofilm is less susceptible to the fungicidal activity of the antifungal drug (P < 0.01). Similarly, voriconazole in combination with cefepime was less active against polymicrobial biofilm compared to the activity against monomicrobial biofilm (P < 0.01). This finding is contrary to what was obtained in the CFU assay where both monomicrobial and polymicrobial biofilms of *A. fumigatus* was almost equally susceptible to voriconazole with and without cefepime. Thus, the apparent resistance of *A. fumigatus* in polymicrobial biofilm to voriconazole may be an artifact of the MTT assay due to the presence of *P. aeruginosa* cells not susceptible to voriconazole but actively contributing to tetrazolium reduction in the polymicrobial biofilms. In support of this suggestion it was noted that a comparison of the effect of voriconazole alone and in combination with cefepime against monomicrobial biofilm is very similar (P > 0.05). Similarly, the effect of voriconazole alone and in combination with cefepime against *A. fumigatus*-*P. aeruginosa* biofilm is almost identical (P > 0.05) showing no significant difference. Thus, since there is no suitable way of separating the fungal and the bacterial contributions to the tetrazolium reduction the MTT assay is unsuitable for studying the bioactivity of voriconazole against *A. fumigatus* biofilm.

**Figure 5:**
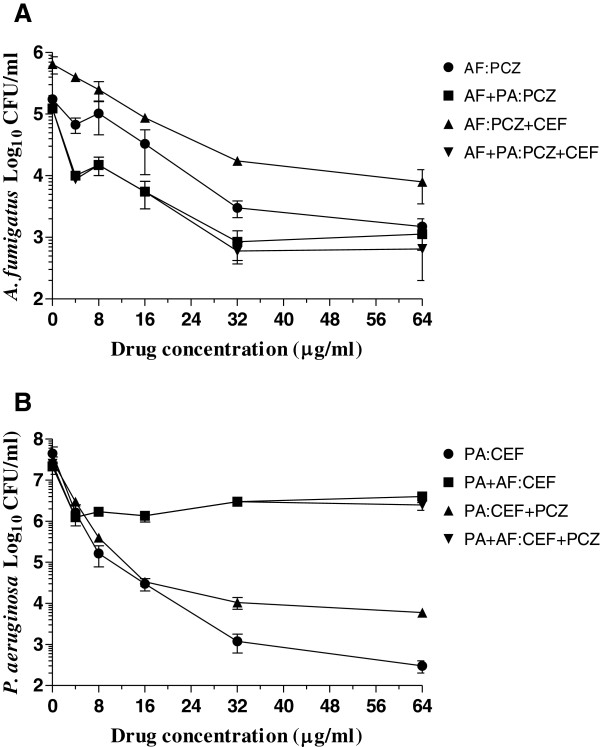
**Biofilm inhibition by posaconazole and cefepime. A**. Effects of posaconazole alone and in combination with cefepime against *A. fumigatus* monomicrobial and *A. fumigatus-P. aeruginosa* polymicrobial biofilms. **B**. Effects of cefepime alone and in combination with posaconazole against *P. aeruginosa* monomicrobial and *P. aeruginosa-A. fumigatus* polymicrobial biofilms. Each experiment was performed two different times with the clinical isolates AF53470 and PA57402 using independently prepared conidial suspensions and bacterial cultures, and one time with the laboratory isolates AF36607 and PA27853. Both clinical and laboratory isolates provided similar results. The data were analyzed by one-way and two-way ANOVA with Bonferroni’s multiple comparison test where each set of data is compared with all the other sets of data as well as by paired two-tailed Student’s *t*-test using Graphpad Prism 5.0. The vertical bar on each data point denotes standard error of the mean for two independent experiments performed with the clinical isolates. **Legends: AF**, *A. fumigatus* monomicrobial biofilm; **PA**, *P. aeruginosa* monomicrobial biofilm; **PA + AF** and **AF + PA**, polymicrobial biofilm; **CEF**, cefepime; **PCZ**, posaconazole.

Figure 5 shows the effects of cefepime and posaconazole individually and in combination on monomicrobial and polymicrobial biofilms of *P. aeruginosa* and *A. fumigatus*. A comparison of the susceptibilities of *A. fumigatus* monomicrobial and *A. fumigatus-P. aeruginosa* polymicrobial biofilms to posaconazole with and without cefepime (Panel A) provided 1 to 1.5 logs CFU reduction at a drug(s) concentration of 64 μg/ml and showed no significant difference (P > 0.05). In contrast, a comparison of the effects of cefepime on *P. aeruginosa* monomicrobial (≈4.5 logs CFU reduction at a 64 μg/ml) and *P. aeruginosa*-*A. fumigatus* polymicrobial (≈1.5 logs CFU reduction at 64 μg/ml) biofilms (Panel B) showed that the polymicrobial biofilm is significantly less susceptible to cefepime (P < 0.0001). Similarly, a comparison of the effects of combination of cefepime with posaconazole on monomicrobial biofilm of *P. aeruginosa* (≈4 logs CFU reduction at 64 μg/ml) with that obtained for polymicrobial biofilm (≈1.5 logs CFU reduction at 64 μg/ml) showed that polymicrobial biofilm is also significantly less susceptible to the combination of drugs (P = 0.0013). However, a comparison of the susceptibility of *P. aeruginosa* monomicrobial biofilm to cefepime alone (≈4.5 logs CFU reduction at a 64 μg/ml) and cefepime plus posaconazole (≈4 logs CFU reduction at 64 μg/ml) showed no significant difference (P = 0.4234) indicating that posaconazole has no detectable effect on the antibacterial activity of cefepime. Similarly, a comparison of the effect of cefepime on polymicrobial biofilm (≈1.5 logs CFU reduction at 64 μg/ml) with that of the combination of cefepime and posaconazole (≈1.5 logs CFU reduction at 64 μg/ml) showed that the polymicrobial biofilm was almost equally susceptible (P = 0.4057) to the drug combination suggesting that the presence of posaconazole in the combination did not affect bioactivity of cefepime against polymicrobial biofilm.

**Figure 6:**
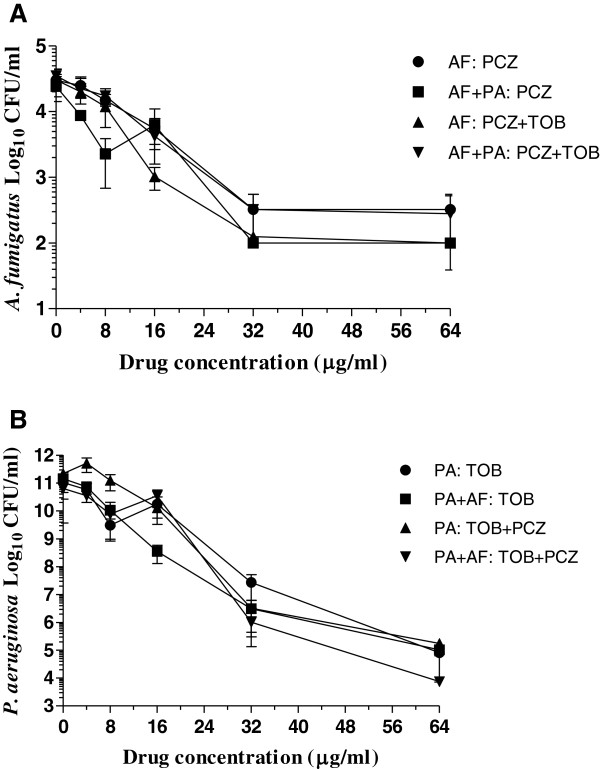
**Biofilm inhibition by posaconazole and tobramycin. A**. Effects of posaconazole alone and in combination with tobramycin against *A. fumigatus* monomicrobial and *A. fumigatus*-*P. aeruginosa* polymicrobial biofilms. **B**. Effects of tobramycin alone and in combination with posaconazole against *P. aeruginosa* monomicrobial and *P. aeruginosa*-*A. fumigatus* polymicrobial biofilms. Each experiment was performed two different times with the clinical isolates AF53470 and PA57402 using independently prepared conidial suspensions and bacterial cultures, and one time with the laboratory isolates AF36607 and PA27853. Both clinical and laboratory isolates provided similar results. The data were analyzed by one-way and two-way ANOVA with Bonferroni’s multiple comparison test where each set of data is compared with all the other sets of data as well as by paired two-tailed Student’s *t*-test using Graphpad Prism 5.0. The vertical bar on each data point denotes standard error of the mean for two independent experiments performed with the clinical isolates. **Legends: AF**, *A. fumigatus* monomicrobial biofilm; **PA**, *P. aeruginosa* monomicrobial biofilm; **AF + PA** and **PA + AF**, polymicrobial biofilm; **PCZ**, posaconazole; **TOB**, tobramycin.

Since cefepime alone and in combination with posaconazole showed differential activity against *P. aeruginosa* monomicrobial and *P. aeruginosa*-*A. fumigatus* polymicrobial biofilms, we investigated the effect of tobramycin alone and in two-drug combination with posaconazole. As shown in Figure 6A, posaconazole with and without tobramycin was almost equally effective against both monomicrobial and polymicrobial biofilms with approximately 2 to 2.5 logs CFU reduction at a drug concentration of 64 μg/ml (P > 0.05). Similarly, Figure 6B shows the effect of tobramycin alone and in combination with posaconazole against *P. aeruginosa* monomicrobial and *P. aeruginosa*-*A. fumigatus* polymicrobial biofilms*.* Tobramycin with and without posaconazole were equally active against the *P. aeruginosa* monomicrobial and *P. aeruginosa*-*A. fumigatus* polymicrobial biofilms with approximately 5-6 logs CFU reduction at a drug concentration of 64 μg/ml (P > 0.05). These results also show that tobramycin and posaconazole has no in vitro drug-to-drug interaction to reduce the bioactivity of the other drug. The excellent activity of tobramycin against monomicrobial and polymicrobial biofilms is in sharp contrast to the differential effects of cefepime alone and in combination with posaconazole against monomicrobial and polymicrobial biofilms of *A. fumigatus* and *P. aeruginosa*.

## Discussion

*P. aeruginosa* is known to produce an array of small molecules possessing antimicrobial activity by direct or indirect interaction with cells. So one of the intriguing questions is why *A. fumigatus* hyphae are refractory to the fungicidal effect of *P. aeruginosa* whereas conidia and sporelings are completely killed. Several reasons could be mentioned for the poor susceptibility of *A. fumigatus* hyphae to the inhibitory effect of *P. aeruginosa* in mixed cultures: (1) Gliotoxin is a cytotoxic compound with antibacterial activity produced by *A. fumigatus*. The synthesis of this mycotoxin molecule is upregulated during mycelial growth in *A. fumigatus*, in particular during biofilm formation. So the increased level of gliotoxin during biofilm formation could inhibit *P. aeruginosa* growth or retards its ability to kill *A. fumigatus*. (2) It is generally known that metabolic activity of the cells is essential for *P. aeruginosa* virulence factors to be effective eliciting its inhibitory action. Germinating conidia and young sporelings are more or less uniformly metabolically active whereas in more mature hyphae metabolic activity is restricted to the apical regions of the filaments where hyphal extension takes place, although any part of growing hyphae is capable of regeneration (pluripotent) producing an actively growing fungal colony. Thus, the metabolically quiescent vegetative mycelia are less susceptible to the cytotoxic molecules produced by *P. aeruginosa*. (3) The cell wall chemistry of the mature hyphae is different from that of the young hyphae and the cell wall of matured hyphae may have restricted permeability to *P. aeruginosa* produced toxic molecules.

*P. aeruginosa* is a well known biofilm producer both in the laboratory and in clinical settings, especially in chronic infections [51-59]. One of the hallmarks of *P. aeruginosa* biofilm is its profound tolerance for antimicrobial drugs and microbiocidal agents while the individual cells of the biofilm community are highly drug susceptible in planktonic cultures [38,40,42,60,61]. Nearly four decades of research has provided a wealth of valuable information on the genesis, architecture, chemical composition and the drug susceptibility of *P. aeruginosa* biofilm [62,63]. In contrast, currently we know very little about *A. fumigatus* biofilm and the first report on *A. fumigatus* monomicrobial biofilm was published by Mowat *et al.*[40,60] in 2007. These investigators described that *A. fumigatus* forms an extensive net work of hyphae producing a multicellular community firmly attached to a solid substrate, and the adherent mycelial growth was encased in an extracellular matrix that resembles a biofilm microbial community. In addition, these investigators described that the extracellular matrix bound adherent fungal cells were highly resistant to antifungal drug treatment [40,60,64] compared to their free-floating counter parts.

The high prevalence [65,66] of *P. aeruginosa* and *A. fumigatus* in CF patients suffering from persistent lung infection provides a highly suitable ecological niche for the production of mixed microbial biofilm. The characteristics of polymicrobial biofilms produced by these organisms in mixed microbial cultures are largely unknown. Thus, the primary objective of our study was to develop a simple reliable easy to perform procedure for the development of a stably adhered polymicrobial biofilm of *A. fumigatus* and *P. aeruginosa* using mixed microbial culture of these organisms.

We examined several types of multi-well cell culture plates (6-well to 96-well) and growth media for the development of *A. fumigatus*-*P. aeruginosa* polymicrobial biofilm in cocultures. Although the 96-well cell culture plate would give a large number of replications for antimicrobial susceptibility studies, the wells in 96-well cell culture plates were found to be too small to prevent cross-contamination between wells by the surface growth of *A. fumigatus*. In contrast, the 6-well and 12-well cell culture plates were found to be too big and comparatively large volumes of medium were needed for the development of biofilms and provided limited number of replications for drug susceptibility studies. In our experience, Costar 24-well cell culture plates were ideal for the development of in vitro monomicrobial and polymicrobial biofilms of *A. fumigatus* and *P. aeruginosa* and provided sufficient number of wells for replications. The large deep wells were adequately separated for multiple manipulations of the biofilm without cross-contamination between wells. In SD broth the 24-h and 48-h mixed microbial cultures of *A. fumigatus* and *P. aeruginosa* produced polymicrobial biofilms at 35°C. Although the biofilm mass was significantly higher in 48 h biofilm, there was no significant difference for the CFU values obtained for the 24-h and 48-h cocultures. Therefore, we would suggest that 24 h growth of the mixed microbial culture will be sufficient to produce a functional *A. fumigatus*-*P. aeruginosa* polymicrobial biofilm for antimicrobial drug susceptibility studies.

The tetrazolium reduction assay has been used by several investigators in the past to examine the viability of a variety of eukaryotic cells ranging from mammalian to fungal cells, including members of the genus *Aspergillus*[48,67-71]. Therefore, we investigated the feasibility of using methyltetrazolium (MTT) assay for monitoring the viability of *A. fumigatus* cells after coculturing with *P. aeruginosa* in mixed microbial biofilms. The MTT assay has been used in our laboratory [68] previously, found to be convenient and highly sensitive for monitoring the viability of *A. fumigatus* cells, in particular after exposure to antifungal drugs. Similarly, we found in the current series of experiments that the MTT assay was very useful for monitoring the viability of *A. fumigatus* cells in monospecies cultures after 24 h and 48 h growth. However, in the mixed species cultures where *A. fumigatus* and *P. aeruginosa* were grown together in cocultures although the assay was highly sensitive and easy to perform, it was found to be difficult to distinguish the contribution made by the bacterial and fungal cells towards the reduction of the MTT compound. Therefore, we used only the CFU assay to monitor the growth of *A. fumigatus* cells in mixed microbial biofilms and for drug susceptibility studies. Apart from the inconvenience, the main disadvantages of using the CFU assay for determining the viability of *A. fumigatus* cells are the under estimation of CFUs due to clumping of hyphae and that the bacteria-treated fungal cells can be inhibited from growing without being killed. Since the end point of CFU assay is the formation of fungal colonies by individual cells, growth inhibition without killing would go undetected. Nonetheless, the fact that we washed the treated cells extensively with sterile distilled water makes it unlikely that in our experiments the fungal cells were only inhibited by the bacterial cells without killing them.

Our results show that the monomicrobial and the polymicrobial biofilms of *A. fumigatus* and *A. fumigatus-P. aeruginosa* were almost equally susceptible to antifungal drugs such as voriconazole and posaconazole. The main reasons for the biofilm to exhibit drug resistance/tolerance are (1) biofilm specific upregulation of efflux proteins (2) the presence of an extracellular matrix and (3) the presence of persistor cells that are inherently drug resistant/tolerant due to their low metabolic rate. It is likely that there is no differential upregulation of efflux proteins in monomicrobial and polymicrobial biofilms of *A. fumigatus* and *A. fumigatus-P. aeruginosa*. Similarly, although it is possible that the extracellular matrix produced by monomicrobial and polymicrobial biofilms of *A. fumigatus* and *A. fumigatus-P. aeruginosa* mixed culture is different, the difference in the permeability characteristics of monomicrobial and polymicrobial biofilm produced extracellular matrices are not sufficient enough to show any reduction in drug penetration. Since the growth characteristics and the biology of *A. fumigatus* is vastly different from other unicellular organisms such as bacteria and pathogenic yeasts, the presence of persistor cells inherently resistant to antimicrobial drug is highly unlikely. Together, these points suggest that although differential antifungal drug susceptibility for *A. fumigatus* monomicrobial and polymicrobial biofilms was expected, the lack of such response is not entirely surprising.

In contrast, our antimicrobial drug susceptibility studies showed that polymicrobial biofilm associated *P. aeruginosa* cells are less susceptible to cefepime in comparison to their monomicrobial counterparts. The extracellular matrix of *P. aeruginosa* biofilm is composed of proteins, polysaccharides, in particular alginate, and eDNA whereas that of *A. fumigatus* biofilm is made up of galactomannan, alpha-1,3 glucans, monosaccharides and polyols, pigments, proteins and eDNA. The most plausible explanation for the reduced susceptibility of polymicrobial biofilm embedded *P. aeruginosa* is the difference in the make up of the extracellular matrix of monomicrobial (*P. aeruginosa*) and mixed microbial (*P. aeruginosa*-*A. fumigatus*) biofilms. The polymicrobial extracellular matrix may have permeability properties different from that of the monomicrobial extracellular matrix preventing adequate access to the biofilm embedded cells.

## Conclusions

The high prevalence of *P. aeruginosa* and *A. fumigatus* colonization of the airways of CF patients results in mixed microbial chronic infections. The polymicrobial CF patient airway infection with *P. aeruginosa* and *A. fumigatus* produces mixed microbial biofilm with structural and functional characteristics different from those of monomicrobial biofilms. The monomicrobial extracellular matrix embedded bacterial and fungal cells are highly resistant to antimicrobial drug therapy. Although the formation of mixed microbial biofilm is considered to be a serious clinical problem in CF patients as well as in other patient groups prone to airway infection with *P. aeruginosa* and *A. fumigatus*, we know very little about the antibiotic susceptibility of *P. aeruginosa-A. fumigatus* polymicrobial biofilm. We therefore investigated the feasibility of developing an in vitro polymicrobial biofilm model using simultaneous static cocultures of *A. fumigatus* and *P. aeruginosa* for studying drug susceptibility. Simultaneous coculturing of *A. fumigatus* conidia with *P. aeruginosa* resulted in the complete killing of the fungus whereas *A. fumigatus* sporelings grown for 12 h or longer were recalcitrant to the fungicidal activity of *P. aeruginosa* and the young hyphae were highly suitable for producing sustainable polymicrobial biofilm with *P. aeruginosa* in cocultures. Using this in vitro model we studied the effects of cefepime and tobramycin alone and combination with posaconazole on monomicrobial and polymicrobial biofilms of *P. aeruginosa* and *A. fumigatus*. Our results show that *P. aeruginosa* cells associated with polymicrobial biofilm were less susceptible to cefepime (but not to tobramycin) compared to those of monomicrobial biofilm. On the other hand, *A. fumigatus* showed similar antifungal drug susceptibility in monomicrobial and polymicrobial biofilms.

## Abbreviations

CF: Cystic fibrosis; SD: Sabouraud’s dextrose; BHI: Brain Heart Infusion; RPMI: RPMI1640; CFU: Colony forming unit.

## Authors’ contributions

**EKM** together with JAV planned and designed all the experiments described in this manuscript, as well as performed the photomicrographic studies, experiments describing the biofilm assay, part of the drug susceptibility studies and the initial data analysis. Also, EKM prepared the initial draft of the manuscript. **DLV** performed all the experiments describing the interaction of germinated and ungerminated *A. fumigatus* conidia with *P. aeruginosa* cells, some of the drug susceptibility experiments as well as the effects of various microbial growth medium on the monomicrobial biofilm formation of *P. aeruginosa* cells on Costar tissue culture plates. **JAV** helped EKM in the planning and designing of all the experiments as well as performed analysis and interpretation of the results. Also, JAV revised the initial draft of the manuscript and prepared the submitted version. All authors read and approved the final manuscript.
